# Sign- vs. goal-tracking is associated with greater adiposity and altered functional connectivity in response to a naturalistic food paradigm

**DOI:** 10.1016/j.physbeh.2025.115075

**Published:** 2025-08-20

**Authors:** Afroditi Papantoni, Grace E. Shearrer, Lindsey Smith Taillie, Saame Raza Shaikh, Katie A. Meyer, Elianna Paninos, Alexxai V. Kravitz, Kyle S. Burger

**Affiliations:** a Department of Nutrition, Gillings School of Global Public Health, University of North Carolina at Chapel Hill, Chapel Hill, NC 27599, USA; b Monell Chemical Senses Center, Philadelphia, PA 19104, USA; c Department of Family and Consumer Sciences, University of Wyoming, Laramie, WY 82071, USA; d Department of Psychiatry, Washington University in St. Louis, St Louis, MO 63110, USA; e Biomedical Research Imaging Center, University of North Carolina, Chapel Hill, NC 27514, USA

**Keywords:** Pavlovian conditioning, Sign-tracking, Goal-tracking, Naturalistic fMRI paradigm, Functional connectivity

## Abstract

Our modern food environment is full of highly palatable, ultra-processed foods that influence our eating behaviors. The reinforcement learning framework posits that some individuals readily assign motivational value to environmental cues (e.g., food ads) that predict reward, biasing their attention and making them more susceptible to seek that reward. These individuals are characterized as sign-trackers and differ from goal-trackers who do not tend to assign any motivational value to those reward-predicting environmental cues. Here, we tested whether this well-characterized phenotype in animals that is commonly associated with increased impulsivity and substance use disorders, could be translated to humans and adapted to study differences in adiposity and eating behaviors. A total of 47 adults completed a food-adapted Pavlovian conditioning task with cues predicting the delivery of candy with simultaneous eye-tracking to determine the sign-tracking vs. goal-tracking phenotype. Participants also completed a naturalistic fMRI scan where they passively viewed videos of sweet and savory dishes to examine functional connectivity associated with those phenotypes. We found that sign-tracking behavior was associated with a greater waist-to-hip ratio but not BMI. During the viewing of the sweet video only, we identified a brain network comprising high-degree nodes in the fusiform gyrus, occipital lobe, prefrontal cortex and posterior cingulate cortex that predicted higher sign-tracking behavior. These findings suggest that the sign- and goal-tracking phenotype model translates to humans using a food-based eye-tracking methodology. Further, supported by animal research, sign-tracking is associated with greater central adiposity and greater functional connectivity between visual, sensorimotor, and subcortical networks.

## 1. Introduction

The current Western food environment is considered to be obesogenic [1,2]; now oversaturated with ultra-processed, highly-palatable foods and food cues designed to encourage overeating. Food choices in the presence of these environmental cues of energy-dense foods (e.g., food images and advertisements, brand logos, smells, etc.) become increasingly complex [3] making it more difficult to decide between consuming a highly-palatable food versus maintaining a healthy diet [4–6]. The constant exposure to these environmental food cues is sufficient to evoke appetitive responses (e.g., food cravings) that motivate us to eat even in the absence of hunger [7]. Thus, it is critical to understand via which neurobehavioral pathways environmental food cues influence food-based decision-making.

One framework used to study how environmental cues reinforce specific food choices is Pavlovian (or classical) conditioning [7–9]. In Pavlovian conditioning, an initially neutral stimulus is paired with a salient unconditioned stimulus (UCS). During repeated exposure to the pairing of the neutral and the unconditioned stimulus, the neutral stimulus becomes conditioned (CS). Subsequently, this CS acquires its own predictive value even without the presence of the UCS and is sufficient to drive the response that was initially associated with the UCS; now referred to as conditioned response [10]. Some Pavlovian responses can be hardwired, but new associations between cues (e.g., restaurant sign) and biologically significant outcomes (e.g., obtaining food) can be learned throughout life [11,12]. Through this Pavlovian valuation system, environmental food cues can induce feeding in humans independent of physiological hunger with the potential to lead to overeating [11–15]. Thus, understanding how this neurocognitive framework influences food choices may reveal novel targets for dietary and pharmacological interventions, as well as inform policy around food marketing.

Sign- and goal-tracking are phenotypes extensively studied in animal research as part of the Pavlovian valuation system to describe how individuals vary in their propensity to assign values to environmental cues (i.e., CS) [16–18]. During the initial Pavlovian learning process, following repeated pairings over time, animals and humans learn the association between the CS and UCS. Following this learned CS-UCS pairing during conditioning, the CS attains a predictive value and elicits a conditioned response. In goal-trackers (GT), the CS acts solely as a predictor of the rewarding UCS and any behavioral response is directly towards the UCS. For sign-trackers (ST) on the other hand, the behavioral response is initially towards the CS, followed by a conditioned response towards the UCS [19,20]. Differences in behavior occur because for GT, the CS has only predictive value, while for ST, the CS attains motivational value, making the CS a salient stimulus of its own that biases attention and reinforces approach behaviors [19,21]. These phenotypes have been studied using the Pavlovian Conditioned Approach test in animals [19,22], with ST showing increased food- and drug-seeking behaviors, greater susceptibility to cue-induced relapse and higher impulsivity and overeating [23–26]. In humans, ST and GT behaviors are usually assessed via Pavlovian conditioning [27–29] and Value-Drive Attentional Capture tasks [30,31]. A recent study using a Pavlovian conditioned approach task in humans, showed that impulsivity was a significant predictor of sign-tracking relative to goal-tracking suggesting that the relationships seen in animal models can be translated for the study of human behaviors [32]. Additional studies indicate that individuals with obesity are more likely to be characterized as ST, where increased attentional bias to rewarding food cues can lead to higher emotional eating and food cravings [21,27,33]. The sign-tracking phenotype exhibits increased resistance to extinction and persists even if the CS becomes associated with an adverse outcome (e.g., bitter taste) or lack of reward [34,35].

Behavioral differences between the two phenotypes are underpinned by differences in the neural processing of Pavlovian learning and attribution of incentive salience. Rodent studies have shown that sign-trackers have higher phasic dopaminergic signaling in the nucleus accumbens, higher amygdala-nucleus accumbens connectivity, and higher thalamus-nucleus accumbens connectivity (for review see [21, 36]). To our knowledge, there are few studies investigating the neural substrates of ST and GT in humans [27,30,37–39], with only two of them studying ST and GT behaviors in response to food rewards [38,39]. One study found that sign-trackers show increased blood oxygen level dependent (BOLD) response in the ventral striatum in response to monetary reward prediction errors, suggesting that dopaminergic signals underlie learning in ST [27]. Additionally, ST behaviors in humans are associated with activation of the attention and salience networks in response to CS paired with monetary rewards [30]. ST (vs. GT) in children is also linked to reduced inferior parietal lobe BOLD activation in response to monetary anticipation (either win or loss), suggesting that ST show less engagement of cognitive control regions when they process the salience of cues [37]. However, a study using electroencephalograms showed that both higher motivational salience to food-related cues and higher cognitive control were independently associated with higher food consumption during the experiment, suggesting that bottom-up motivational processes and top-down cognitive control processes independently contribute to the regulation of cue-induced eating in humans [38].

Collectively, there is some evidence that the ST and GT phenotypes exist in humans and might play a role in how different individuals attribute incentive salience to environmental cues, which putatively puts sign-tracking individuals at higher risk for compulsive behaviors. Given that most of this evidence comes from the study of addiction and substance use in animal models as well as the study of monetary rewards in humans, we investigated if ST/GT behaviors can be replicated using food-specific rewards (UCS) and whether these behaviors are associated with adiposity and differences in brain functional connectivity during the passive viewing of a food show. We hypothesized that ST would have higher adiposity and show increased functional connectivity between subcortical, attention, and salience networks.

## 2. Methods

### 2.1. Sample & study overview

Sixty-nine (*n* = 69) participants were recruited from the Chapel Hill area in North Carolina, between 2022 and 2023 to complete a cross-sectional study that included one study visit and one optional follow-up visit. Inclusion criteria included: 1) age 18–45 years, 2) body mass index (BMI; kg/m^2^) between 18.5 and 40. Exclusion criteria were: 1) contraindications of MRI (e.g. metal implants, pregnancy, pacemakers), 2) self-reported current or past diagnoses of an eating disorder, 3) medication use that could affect appetite and weight (e.g. metformin, levothyroxine, GLP-1), 4) allergy or intolerance to study foods, 5) diagnosis of a major psychiatric disorder (schizophrenia, bipolar disorder). Individuals who reported a diagnosis of depression or anxiety and reported taking an antidepressant or anti-anxiety medication without a dosage change within the last 3 months were considered eligible. The Institutional Review Board of the University of North Carolina at Chapel Hill approved all methods and study participants gave written consent before the start of the study. Out of the 69 participants enrolled, 2 were excluded because they were ineligible (1 participant had a BMI outside the inclusion range, 1 participant got pregnant during the study), 16 did not complete the optional follow-up, 1 could not complete the MRI scan due to unforeseen contraindications, 1 participant was excluded due to technical problems with the study equipment, and 2 were removed due to poor valid gaze data during the eye-tracking task resulting in an analytic sample of 47 participants. In this analytic sample, 5 participants (10.6 %) reported taking an antidepressant/anti-anxiety medication without a dosage change in the last 3 months (e.g., sertraline, Well-butrin, Lexapro, venlafaxine). All analyses reported below were repeated with a variable representing this medication usage.

Participants came in for one baseline and an optional follow-up study visit. Participants were instructed to fast for 3 h prior to the baseline visit and 4 h prior to the optional follow-up. The baseline study visit took place at the UNC School of Medicine Biomedical Research Imaging Center. The optional follow-up visit took place at the UNC Center for Health Promotion and Disease Prevention. The anthropometrics and MRI data described below were collected during the baseline visit, while the Pavlovian conditioning task was completed during the optional follow-up visit. Fasting blood glucose and hemoglobin A1C was collected at both visits. A detailed overview of the study visits can be seen in Supplementary Figure 2.

### 2.2. Anthropometrics and demographics

Height was measured to the nearest 0.1 cm using a stadiometer and weight was measured to the nearest 0.1 kg using a digital scale with participants wearing light clothing without shoes. BMI (kg/m^2^) was then calculated. Waist circumference was measured to the nearest 0.1 cm at the approximate midpoint between the lowest rib and the top of the iliac crest. Hip circumference was measured to the nearest 0.1 cm at the widest part of the hips. Waist-to-hip ratio (WHR) was then calculated. Height, weight, waist, and hip circumference measurements were repeated twice for each participant, and the average was used for further analysis. Participants also completed a brief survey to collect information on sex assigned at birth, race/ethnicity, current medication use and food security status (assessed via the USDA Six-Item Food Security Scale [40]).

### 2.3. Fasting blood glucose and hemoglobin A1C

Fasting blood glucose was measured using an Accu-Check Aviva Plus Glucose Meter (Roche Diabetes Care, Inc, Indianapolis, IN) that provides immediate results. Hemoglobin A1C (HbA1C) was measured at the same time using the A1CNow^®^+ test kit (pts Diagnostics, Whitestown, IN). Measurements occurred in both visits.

### 2.4. Pavlovian conditioning task

Participants completed a modified food-based version of the Pavlovian-to-Instrumental Transfer (PIT) task adapted from [27,41] to assess Pavlovian conditioning (PsychoPy v2022.1.1). The PIT task consists of four parts (Pavlovian conditioning, Instrumental conditioning, Pavlovian-to-Instrumental transfer, Forced choice) but only the first is described below as it is used to assess sign-/goal-tracking behavior. For details on the full PIT task see Supplementary Materials and Supplementary Figure 1. Eye-tracking (Tobii Pro Glasses 3; Tobii Pro AB, Stockholm, Sweden) was used during the Pavlovian conditioning part to assess ST/GT behavior. A candy dispenser was used during the Pavlovian conditioning task to deliver the rewarding food stimulus. The candy dispenser, designed and built by Dr. Kravitz, used the Adafruit Feather M4 express for control, and a ULN2003 Feather board to control the dispensing motor. Further information on the candy dispenser, including assembly files, can be accessed at: https://hackaday.io/project/174102-mm-dispenser-for-research-use-only.

#### Pavlovian conditioning:

**Fig. 1.**
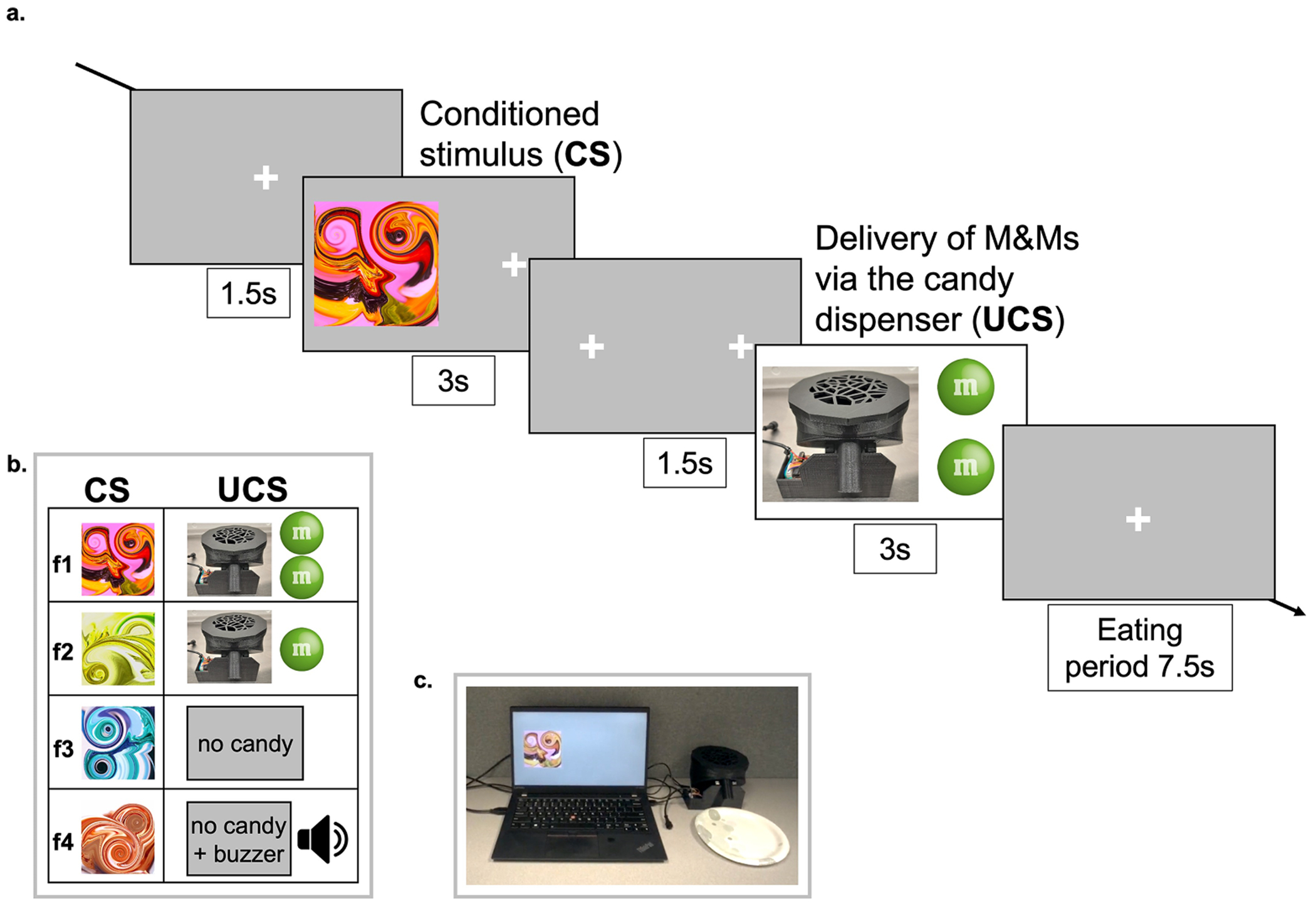
The Pavlovian Conditioning Task. **(a)** Example trial from the Pavlovian conditioning task [adapted from Schad et al. 2020]. In each trial participants were shown one of four possible fractal CSs. The fractal CSs were paired with either food rewards (2 candies, 1 candy) or no rewards (no candy, no candy + buzzer sound). The task had 8 shaping trials and 64 (16 per fractal CS type) experimental trials. Eye-tracking data were collected during this part. Task duration was 20 min. **(b)** List of the four CS-UCS pairings. These pairings were fixed for all participants. **(c)** Actual task setup.

The Pavlovian conditioning task (Fig. 1) included 4 distinct fractal images (f1, f2, f3, f4) that served as the conditioned stimuli (i.e., ‘fractal CS’), each paired with a different UCS (2 candies; 1 candy; no reward; no reward + buzzer sound). The CS-UCS pairings were fixed for all participants. At the beginning of each trial, a fixation cross was presented in the middle of the screen for 1.5 s. The CS was presented for 3 s on the left side of the computer screen, followed by two fixation crosses presented on the left and right side of the screen (1.5 s). The UCS was then delivered from the candy dispenser on the right side of the computer screen (opposite of the fractal CS; 3 s). This was followed by 7.5 s of fixation, where the participant consumed the candy and reset their attention and hands. The first 8 trials presented were shaping trials, where the CS-UCS pairings were presented first in descending order (2 pieces of candy, 1 piece of candy, no reward, no reward + buzzer sound) and then in ascending order (no reward + buzzer sound, no reward, 1 piece of candy, 2 pieces of candy). Following the first 8 shaping trials, all participants completed 64 trials consisting of 16 presentations of each CS-UCS pairing. The four pairings were presented in pseudorandomized blocks of four, ensuring each pairing appeared once per block before any repetition occurred. After 32 trials, the orientation of the monitor and candy machine were switched, such that the fractal CS appeared on the right side of the computer screen and the machine delivered candy on the left side of the monitor. Participants were instructed to observe the fractal CS and the candy delivery and to memorize the pairings. The participants were told to consume the candy by grabbing it with their dominant hand otherwise have their dominant hand placed on the table just below the monitor. They were also told that when the orientation was switched there was no effect on the relationship between the fractal pictures and the candy and that we were simply moving the stimuli around. Regular M&M^®^ chocolate candies were used as the rewarding UCS.

Prior to the task, participants rated their hunger on a visual analog scale (VAS) anchored by – 100 (least hungry imaginable) to 100 (most hungry imaginable). After the task, participants rated the pleasantness and desirability of the M&M candies consumed during the Pavlovian conditioning task via VAS, anchored by – 100 (least pleasant/desirable imaginable) to 100 (most pleasant/desirable imaginable).

### 2.5. Behavioral data statistical analysis

Statistical analyses for all behavioral data were performed in R (version 4.3.1, The R Foundation for Statistical Computing). We used a series of separate linear regression models to assess how the sign-/goal-tracking regression coefficient (as calculated below in Section 2.6) is associated with 1) BMI, and 2) WHR. All models were adjusted for age, sex and pre-task hunger ratings.

### 2.6. Sign- and goal-tracking analysis

#### Eye-tracking:

During Pavlovian conditioning, eye position and pupil size were collected using a pair of Tobii Pro 3 eye tracking glasses (Tobii Pro AB, Stockholm, Sweden). The glasses had a sampling rate of 50 Hz, i. e., they register the position of the eyes 50 times per second or once every 20 milliseconds. Calibration of the glasses was performed right before the start of the Pavlovian conditioning and subsequently as needed in the event of the glasses getting disconnected during the 20-minute task (occurred 12 times across the entire analytic sample). For calibration, participants were instructed to focus their gaze on the center of a handheld calibration card. Eye-tracking recordings of the Pavlovian conditioning task acquired with the Tobii Pro Glasses 3 were analyzed using the Tobii Pro Lab software v1.232. The eye-tracking recording spanned the duration of the Pavlovian conditioning task, but due to participant blinking or looking outside the glasses frame, the percentage of correctly identified raw eye movements by the eye-tracker (i.e., gaze sample) over the entire task duration varied per participant. The mean gaze sample in our analytic sample during the entire task was 93.4(4.3) % (range: 84–100 %). Two participants with gaze samples of 62 % and 71 % were removed from any further analysis as mentioned above.

Raw eye movement data were processed by applying an eye movement classification algorithm, or gaze filter, to the data to classify eye movements into 1) fixations, 2) saccades, or 3) any other eye movement. The classification algorithm used here was the Tobii I-VT Fixation Filter, which classifies eye movements based on the velocity of the directional shifts of the eye [42]. If the velocity of the eye movement is below a certain threshold, the samples are classified as part of a fixation. If the velocity is above the threshold, it is classified as a saccade. The velocity threshold for valid fixations was set at 30°/s, with minimum fixation duration set at 50 ms. Only eye movements classified as fixations were considered valid gaze data and used in assessing sign- and goal-tracking behavior.

#### Percent gaze fixations:

**Fig. 2.**
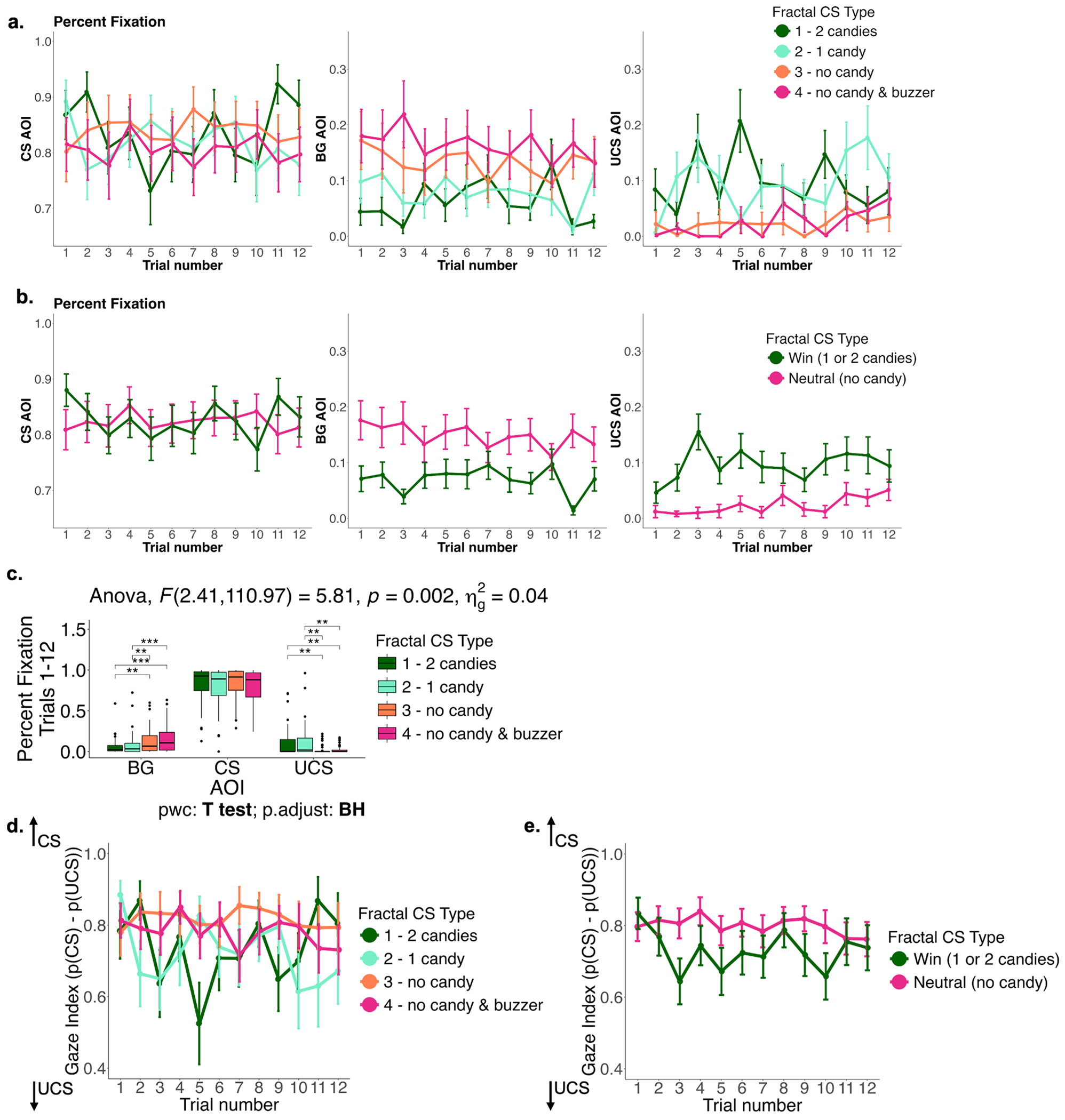
Percent gaze fixation and gaze index during the eye-tracking assessment. Percent fixation per AOI (CS, BG, UCS) for **(a)** each fractal CS type and **(b)** averaged across win (or rewarding) fractals (fractals 1 & 2) and across neutral fractals (fractals 3 & 4). **(c)** Repeated-measures ANOVA comparing percent fixations by AOI and fractal CS type, with a significant AOI by fractal CS type interaction (F(2.41,110.97)=5.81,*p* = 0.002), such that for the BG AOI, percent fixation is higher for the neutral fractals (3 & 4) compared to the rewarding fractals (1 & 2), while for the UCS AOI, percent fixation is higher for the rewarding fractals compared to the neutral ones (*p* < 0.01 across significant pairwise comparisons). Gaze index for **(d)** each fractal CS type and **(e)** averaged across win (or rewarding) fractals (fractals 1 & 2) and across neutral fractals (fractals 3 & 4) displayed per trial. AOI: Area of interest, BG: background, CS: conditioned stimulus, UCS: unconditioned stimulus.

Valid gaze fixations during the entire task duration were manually classified by two independent researchers as being directed at one of 3 spatial areas of interest (AOIs): 1) the fractal CS, 2) the UCS (i.e., the spatial location of the candy dispenser), and 3) background (BG; rest of the visual field). As described above and shown in Fig. 1, the Pavlovian conditioning task included 4 unique fractal CS types, each associated with a different UCS (fractal CS 1 paired with 2 candies, fractal CS 2 paired with 1 candy, fractal CS 3 paired with no reward, fractal CS 4 paired with no reward + buzzer sound), with 16 trials per CS type (64 trials in total). During each trial, the CS was presented on screen for 3 s. In order to measure Pavlovian conditioned responses, following the methods described in [27], we focused our analysis on the last 1.5 s of the fractal CS presentation. CS onset is believed to attract attention and trigger an initial orientation response [43,44] independent of sign- or goal-tracking phenotypes [45], with Pavlovian conditioned responses being established during later stages of CS presentation [43,44,46]. Prior to further analysis, we excluded the last 4 trials for each CS type, due to poorer data quality compared to previous trials (possibly due to participant fatigue and increased body and head movements that lead to suboptimal calibration and data acquisition), thus leaving us with 12 trials per CS type (48 trials in total). Further details on the differences between the included 48 trials and the excluded last 16 trials as well as additional quality control measures can be found in Supplementary Materials and Supplementary Figure 3. The percent of valid gaze fixations on the CS AOI, the UCS AOI and the BG AOI were calculated separately during the last 1.5 s of CS presentation. These variables represent the relative time each participant spent approaching (or fixating on) the CS, UCS and background, respectively. In addition to calculating the percent gaze fixations separately for each CS type, we averaged across fractal CS 1 and 2 (positive/rewarding CS) and fractal CS 3 and 4 (neutral CS). Percent gaze fixations are shown in Figs. 2a,b. We used repeated measures ANOVA to test how percent fixations on each AOI differed by CS type, with a significant AOI by fractal CS type interaction (F(2.41,110.97)=5.81,*p* = 0.002), such that for the BG AOI, percent fixation is higher for the neutral fractals (3 & 4) compared to the rewarding fractals (1 & 2), while for the UCS AOI, percent fixation is higher for the rewarding fractals compared to the neutral ones (*p* < 0.01 across significant pairwise comparisons; Fig. 2c). Additionally, we performed random-effects linear regression analyses to test how percent fixations changed over time, by regressing percent fixations on trial number separately for each AOI. We found that over time, percent fixation on the UCS AOI increased (*p* = 0.039), driven by increases for fractal CS 2 (1 candy; *p* = 0.013) and fractal CS 4 (no candy + buzzer; *p* = 0.002). There was no significant effect of time (i.e., trial number) on the other two AOIs (CS and BG).

#### Gaze index:

The gaze index was calculated per participant as the difference between percent fixation on the CS AOI minus percent fixation on the UCS AOI during the last 1.5 s of CS presentation (*p*(CS) – *p* (UCS)). The gaze index here is reported as a proportion instead of a percent to match previous methodologies in animal and human research [27,47], with a possible range of −1 to 1. A gaze index of 1 indicates that the entire valid fixation time is spent looking within the CS AOI. A gaze index of −1 indicates that the entire time is spent looking within the UCS AOI. In addition to calculating the gaze index separately for each CS type, we averaged across the two positive/rewarding CS and the two neutral CS. Gaze index values are shown in Figs. 2d,e. We performed random-effects linear regression analyses to test how the gaze index changed over time separately for each CS type. The gaze index for fractal CS 2 (1 candy) decreased over time (*p* = 0.039) but no other significant effects of time (i.e., trial number) were observed.

#### *Characterization of sign*- vs. *goal-tracking behavior*:

**Fig. 3.**
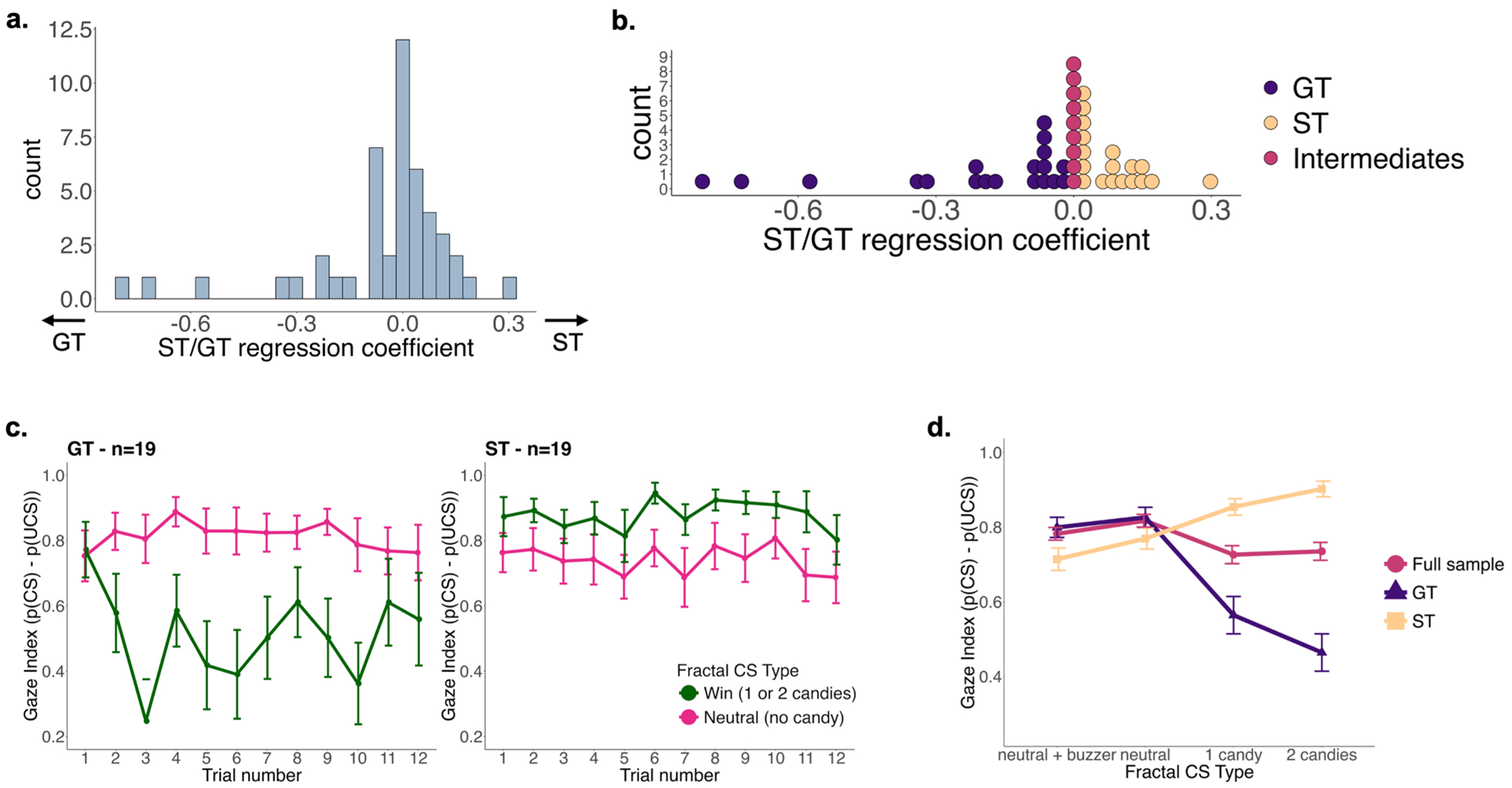
Distribution of the ST/GT CS value regression coefficient and eye-tracking assessment by group (ST and GT). **(a)** Distribution of the ST/GT regression coefficient, with higher values representing more ST behavior (i.e., subjects that tended to fixate on the CS during the rewarding trials). **(b)** Distribution of the ST/GT regression coefficient where the 2/5^ths^ with the most positive regression coefficient were defined as sign-trackers (ST; *n* = 19), the 2/5^ths^ with most negative regression coefficient were defined as goal-trackers (GT; *n* = 19), and the middle 1/5^th^ was defined as an intermediate group (*n* = 9). **(c)** Gaze index for GT and ST averaged across win (or rewarding) fractals (fractals 1 & 2) and across neutral fractals (fractals 3 & 4) displayed per trial. **(d)** Gaze index for the GT group, ST group, and full sample averaged across all trials displayed per fractal CS type. GT: goal-trackers, ST: sign-trackers, CS: conditioned stimulus, UCS: unconditioned stimulus.

To define sign-tracking vs. goal-tracking behavioral tendencies, first we assigned a value on the fractal CS based on the actual reward received. Fractal CS 1 value was 2, fractal CS 2 value was 1 and fractal CS 3 and 4 both had a value of 0 as they represented neutral stimuli with no candy reward. Then we regressed the gaze index on the value of the CS. Participants with a more positive regression coefficient were characterized as showing more of a sign-tracking behavior, as their gaze was attracted more towards the fractal CS predictive of candy rewards. Participants with a more negative regression coefficient were characterized as showing more of a goal-tracking behavior, as their gaze was attracted more by the actual “goal”, i.e., the UCS (here the candy dispenser + the candy). The distribution of the regression coefficient can be seen in Fig. 3a. This ST/GT regression coefficient was used as a continuous variable to test associations between sign- and goal-tracking behaviors with body composition measurements (BMI, WHR), and brain functional connectivity as described below. In post-hoc analyses, the 2/5^ths^ with the most positive regression coefficient were defined as sign-trackers (*n* = 19), the 2/5^ths^ with most negative regression coefficient were defined as goal-trackers (*n* = 19), while the middle 1/5^th^ (*n* = 9) were labeled as intermediates and excluded from the post-hoc group analyses. The distribution of the CS value ST/GT regression coefficient and the gaze index in the sign-trackers and goal-trackers can be seen in Figs. 3b–d.

### 2.7. MRI data acquisition

Anatomical and functional imaging data were collected in a Siemens Prisma 3T scanner (Siemens Medical Solutions, Munich, Germany). Due to software upgrades and repairs, two different Siemens Prisma 3T scanners were used to acquire MRI data and the MRI scanner used was added as covariate in relevant analyses. Functional images were obtained using a T2*-weighted echo planar imaging (EPI) pulse sequence with TR = 2010 ms, TE = 23 ms, flip angle = 80°, matrix = 72 × 72, FOV = 216 × 216 × 108 mm^3^, 36 slices with interleaved acquisition, slice thickness = 3 mm, and voxel size 3 × 3 × 3 mm^3^. Images were acquired along the AC-PC transverse, oblique plane as determined by the midsagittal section. Anatomical images were obtained using a T1-weighted magnetization-prepared rapid-acquisition gradient echo (MPRAGE) sequence with TR = 2400 ms, TE = 2.24 ms, flip angle = 8°, matrix = 320 × 300, FOV = 256 × 240 × 167 mm^3^, slice thickness = 0.8 mm, and voxel size 0.8 × 0.8 × 0.8 mm^3^.

### 2.8. fMRI naturalistic food show scan paradigm

Participants completed two 8 min 48 s runs where they passively watched food cooking videos with audio. One run included a video of a man making brownies (“sweet” run) and the other included a video of the same man making a chicken casserole (“savory” run). Order of videos across participants was randomized. The clips were downloaded from YouTube, both by the same creator [48,49]. Both savory and sweet videos had the same background/setting, same duration (8:34 min for savory; 8:33 min for sweet) and were presented by the same individual. Both videos had a similar number of spoken words with the savory video averaging 184 words/min and the sweet 192 words/min. The sweet video had slightly cooler lighting.

### 2.9. fMRI data preprocessing

Neuroimaging data were preprocessed using the fMRIPrep 20.2.7 pipeline [50]. Prior to preprocessing, DICOMS were converted to the Brain Imaging Data Structure (BIDS) format [51] and the first 4 volumes of each functional run were trimmed. Anatomical data preprocessing included skull stripping using Advanced Normalization Tools (ANTs); brain tissue segmentation using FSL’s (FMRIB Software Library, www.fmrib.ox.ac.uk/fsl) Automated Segmentation Tool (FAST); volume-based spatial normalization to Montreal Neurological Institute (MNI) 152-Asymmetrical space through nonlinear registration using ANTs. Functional data were corrected for field map distortions and co-registered to the anatomical data using FSL’s FMRIB’s Linear Image Registration Tool (FLIRT) with boundary-based registration. Functional data were slice-time corrected using Analysis of Functional NeuroImages (AFNI), with slices realigned in time to the middle of each TR. Functional data were additionally motion corrected using FSL’s MCFLIRT and resampled into MNI 152-Asymmetrical space.

### 2.10. fMRI data analysis

#### Functional connectivity:

Following fMRI preprocessing, we used a whole-brain functional atlas defined on a separate group of healthy adult subjects [52] to parcellate the brains of each subject into 268 regions of interest (ROIs). We chose the Shen 268-node parcellation because it covers the whole brain, including subcortical areas and cerebellum and has been used in previous connectome-based predictive modeling (CPM) studies [53–56]. Here, the mean timecourses per run per participant (i.e., average BOLD signal of all voxels within the node during each task run) for each of the 268 nodes were extracted using Nipype 1.8.6 [57] implemented in Python 3.11.2. Timecourses were extracted separately for the “sweet” and “savory” runs, standardized within subject and detrended to remove linear trends from the signal. In addition, the following 16 confounds were included: 6 motion parameters and their first-order derivatives, global signal, CSF signal, white matter signal, and framewise displacement. To further control for motion, volumes with framewise displacement >0.5 mm were flagged as spikes and regressed out. A functional run for a subject was excluded if >25 % of total volumes were flagged as high motion spikes. Functional connectivity matrices for each subject were created by correlating each node timecourse with every other node timecourse to construct 220 × 220 matrices, one per subject (48 nodes in the cerebellum were removed due to inadequate brain coverage during fMRI acquisition; note that other notes that fall into the cerebellar canonical network – for instance nodes in the brainstem – were kept in). Correlation coefficients in the 220 × 220 connectivity matrices were transformed to z-scores using Fisher’s transformation.

#### Connectome Based Predictive Modeling (CPM):

CPM is a data-driven approach that uses whole-brain functional connectivity to develop models of brain-behavior relationships using machine learning [58,59]. CPM analysis was conducted using previously validated custom scripts in MATLAB [59,60]. A detailed description of the CPM protocol can be found in [59]. An overview of the method is as follows:

- Data, comprising each subject’s functional connectivity matrix and behavioral score (here the gaze index regression coefficient), were divided into training and test sets for leave-one-out cross-validation (LOOCV). We used LOOCV following the current recommendations for validating predictive models in smaller sample sizes [61]. In LOOCV, the training set includes all but one observation, which is used as the testing set. This process is repeated as many times as the sample size, leaving a different observation out of the training set each time. We ran two separate CPM models, one with the “sweet” run functional connectivity and one with the “savory” run functional connectivity.
- In the training set, each edge in the connectivity matrix was correlated with the target behavioral variable (i.e., the sign-/goal-tracking CS regression coefficient calculated above). Target variables were not normally distributed; thus, we used partial Spearman’s rank correlation to control for age, biological sex, run order (i.e., “sweet” or “savory” first), pre-scan hunger ratings, WHR and scanner.
- A feature selection threshold was applied to select the most relevant edges for use in the predictive model based on the correlation coefficients calculated in (b). Here, we retained edges with |rho|>0.3 (corresponding to a two-tailed p-value of approximately 0.04) for the correlation between connectivity and the target variable. Edges were separated into a positive network (edges where connectivity is positively correlated with target variable) and a negative network (edges where connectivity is negatively correlated with target variable).
- For each subject in the training set, we calculated the summed connectivity strength across all retained edges in the positive and negative networks separately.
- Still using the training set only, we fitted two linear models, one using the positive network and the other using the negative network, by regressing network strength on the target behavioral variable. The model parameters were extracted and saved.
- For each subject in the test set, we calculated positive and negative network strength using the same method described in (c). These values were used as input to each of the linear models estimated in (e) to create the predicted target behavioral scores for each subject.

To assess prediction accuracy of each linear model (positive and negative), we correlated the predicted (model generated) and observed target behavioral variables across subjects using Spearman’s rank correlation (to account for the nonnormal distribution of the gaze index regression coefficient). In cross-validation, regression folds are not independent of one another, as such, we used non-parametric permutation testing to assess the statistical significance of prediction accuracy. We generated a null distribution for the correlations between the predicted and observed behavioral scores by randomly shuffling the target behavioral variable with respect to connectivity matrices and repeating the entire CPM pipeline 10,000 times. The non-parametric p-value for each network strength was calculated as the proportion of permuted correlation coefficients (i.e., from the null distribution) that are greater than or equal to the true prediction correlation coefficient (i.e., mean across all the true models) for that network strength model. For data visualization, edges selected in at least 90 % of all folds across all 100 iterations were extracted and graphed for the positive and negative networks separately. Figures were constructed using BioImage Suite [62]. The 220 nodes included in the analyses were assigned to ten canonical networks as defined in [63,64] emphasizing different properties of the human brain and consist of medial-frontal (MF) fronto-parietal (FP), default mode network (DMN), motor/somatosensory (Mot), primary visual (VI), secondary visual (VII), visual association (VAs), salience (SAL), subcortical (SC) and cerebellar (CBL) networks.

## 3. Results

### 3.1. Participant characteristics

Participant characteristics for the analytic sample (*n* = 47) are summarized in Table 1. The analytic sample consisted of 16 (34 %) male and 31 (66 %) female healthy adults (age=25.3 ± 5.8 years; BMI=25.2 ± 3.6 kg/m^2^). When comparing the analytic sample (*n* = 47) with those not included in the analysis (*n* = 22, exclusion as described in Section 2.1), there were no differences in age, BMI, WHR, sex, and food security status, but there was a significant difference in race/ethnicity distribution such that the percent of participants who identified as White was higher in those included vs. not included in the analytic sample (*p* = 0.021). Participants arrived fasted for both the baseline visit and the optional follow-up. Mean self-reported fasting times were 5 ± 2 h for baseline and 6 ± 2 h for the follow-up visit. The time between the two visits was 34±42 days. Mean fasting blood glucose was 88±6 mg/dL for the baseline visit and 90±6 mg/dL for the follow-up. 21 (44.7 %) of participants consumed all the M&M candies disbursed from the machine, 7 (14.9 %) consumed approximately ¾ of all candies disbursed, 8 (17 %) consumed approximately ½ of all candies, 10 (21.3 %) consumed ¼ of all candies, and 1 (2.1 %) participant did not consume any candy. Candies consumed were not related to the ST/GT regression coefficient (*p* = 0.654). The pleasantness and desirability VAS ratings of the candies did not correlate with the regression coefficient (pleasantness *r*=−0.129, *p* = 0.400; desirability *r*=−0.200, *p* = 0.187). Pre-task hunger did not correlate with the regression coefficient (*r*=−0.045, *p* = 0.765). Participant characteristics by ST and GT groups can be seen in Supplementary Table 1. Participants in the ST group were significantly older (*p* < 0.001).

### 3.2. ST/GT and adiposity

The sign-/goal-tracking regression coefficient was significantly associated with WHR (*b* = 1.48, 95 % CI = (0.13, 2.84), *p* = 0.033; Table 2 and Supplementary Figure 4), such that propensity for ST was associated with higher WHR. No association with BMI was observed (*b* = 0.01, *p* = 0.529; Table 2). Results remained similar in significance when controlling for medication use (WHR: *b* = 1.47; *p* = 0.036; BMI: *b* = 0.01, *p* = 0.499). To confirm these findings, we performed post-hoc analyses comparing WHR and BMI between the ST and GT groups using two-sample *t*-tests. WHR was significantly higher in the ST compared to the GT group (*t* = 2.38, df=27.3, *p* = 0.024), but no differences were seen between the groups for BMI (*p* = 0.14; Supplementary Figure 5).

### 3.3. ST/GT and functional connectivity

**Fig. 4.**
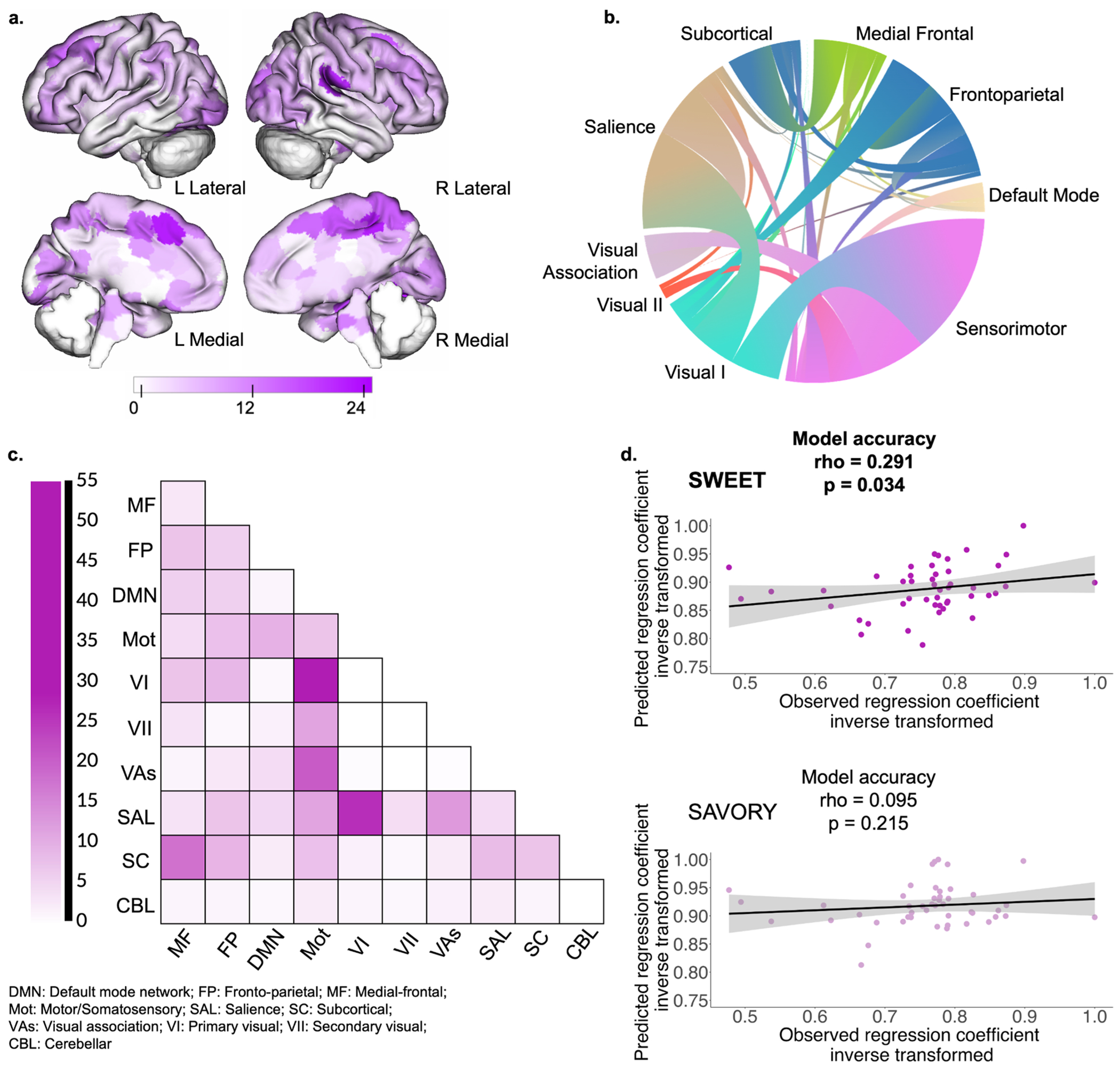
Brain networks associated with the ST/GT regression coefficient during passive viewing of the sweet food show. Representation of positive ST/GT networks during passive viewing of the sweet food show. Greater number of connections between the displayed nodes is associated with larger ST/GT regression coefficient (i.e., more sign-tracking-like behavior). Darker colored regions (**a**) indicate nodes with a greater number of connections. Chord diagrams (**b**) represent the connections among the ten canonical brain networks. Matrix cells (**c**) represent the total number of edges connecting nodes between each network pair among the canonical networks, where darker colors indicate more connections. Scatterplots (**d**) represent model accuracy, i.e., the relationship between the observed versus the predicted ST/GT regression coefficient generated by the functional connectivity CPM models during the passive viewing of the sweet (significant model) and savory (non-significant model) food shows. P-values were calculated using non-parametric permutation testing. Models have been adjusted for age, biological sex, run order (i.e., “sweet” or “savory” first), pre-scan hunger ratings, WHR and scanner.

Using connectome predictive modeling, we examined whether functional connectivity in response to each type of the naturalistic food show paradigm was significantly associated with the sign-/goal-tracking regression coefficient. This was analyzed separately for the sweet and savory runs. We identified a functional connectivity network in response to the passive viewing of the sweet food show where stronger connectivity among the nodes of the network was associated with a higher sign-/goal-tracking regression coefficient and thus with propensity toward sign-tracking behavior (rho=0.291, permutation *p* = 0.034; Fig. 4). This “positive” network contained 1210 edges (5.0 % of all possible connections among the nodes in the atlas used). Highest-degree nodes (i.e., nodes with the greatest number of nodal connections contributing to the network) were found in the fusiform gyrus, occipital cortex, prefrontal cortex, and dorsal posterior cingulate cortex (Table 3). These nodes are summarized based on their overlap with the ten canonical networks and can be seen in Figs. 4b,c. We observed connections between the salience and primary visual networks, between the sensorimotor and primary visual networks, as well as between the subcortical and medial-frontal canonical networks.

No significant networks were detected in response to the savory food show (rho=0.095, permutation *p* = 0.215). Results remained similar in significance when controlling for medication use (sweet: rho=0.289, permutation *p* = 0.033; savory: rho=0.069, permutation *p* = 0.252).

## 4. Discussion

In this study, we used a novel eye-tracking Pavlovian food conditioning task to assess sign- and goal-tracking behaviors in humans and evaluate their neural correlates during exposure to naturalistic food videos. Supported by preclinical research, we showed that increased sign-tracking behavior was associated with greater central adiposity and greater functional connectivity within a network comprising nodes in the fusiform gyrus, occipital cortex, prefrontal cortex, and dorsal posterior cingulate cortex. To our knowledge, this is one of the few studies in humans using food as the rewarding UCS stimulus, as most studies utilize monetary rewards. These findings highlight the successful animal-to-human translation of a method for assessing sign- and goa-tracking phenotypes and suggest that sign-tracking is associated with increased engagement of brain regions in the visual, salience, subcortical and mediofrontal networks.

Previous research has consistently shown that obesity-prone rats are more likely to exhibit sign-tracking behaviors compared to obesity-resistant rats [65] and sign-tracking is linked to increased vulnerability to substance use [66]. However, in humans, results appear more complex. A meta-analysis showed that attentional bias towards food cues – an indirect measure of attributing incentive salience to cues akin to sign-tracking behavior [36] – was not associated with BMI, but instead, it correlated with food intake, subjective food cravings and hunger [67]. Our findings are in line with that review, as BMI was not related to food-specific sign-tracking. Instead, we found that a measure of central adiposity – waist-to-hip ratio – is linked to higher sign-tracking behavior towards food cues. This discrepancy between BMI and WHR has been observed in studies of other behaviors and health outcomes, including all-cause mortality [68,69] and fast food consumption [70], suggesting that general and abdominal adiposity offer insights even independent of each other [69]. Nonetheless, there have been studies in humans showing that Pavlovian conditioning to hedonic food cues is more prevalent in individuals with higher BMI in similarly sized samples and similar age groups [33,71] as presented in this study. This highlights the need for well-powered studies and more granular measures of adiposity, percent body fat and fat composition beyond just BMI. Notably, while not statistically significant, our findings when using BMI were in the same direction as the WHR results, suggesting a weaker, but similar effect. A recent review of the evidence between obesity and attribution of incentive salience to food cues seen in sign-tracking suggests that the association between the two might be primarily driven by increased exposure and consumption of a high-fat high-sugar palatable diet and not by the actual weight gain [72]. This finding supports the need for more studies using food-specific Pavlovian conditioning tasks to study these phenotypes with respect to other behavioral variables beyond adiposity (e.g. dietary exposure). Interestingly, in this sample we saw that age was a significant predictor for the ST/GT regression coefficient with older individuals exhibiting more sign-tracking behavior during the Pavlovian conditioning task. Existing studies in humans have not explicitly tested or reported any association between age and sign-tracking behavioral phenotypes [27–33]. Given participants’ responses to the final part of the PIT task (“Forced choice” depicted in Supplementary Figure 1), we know that older participants had overall slower reaction times with no differences in response accuracy (results not reported here). As such, we could hypothesize that older individuals were generally slower in their reactions hence they kept their gaze fixated on the CS stimulus longer (which would characterize their behavior as sign-tracking). Other explanations for the effect of age are plausible (e.g., perhaps this behavioral phenotype is dynamic and chronic exposure to environmental predictive cues makes us more susceptible to assign motivational value to them) but more studies would be required to replicate and explain this finding.

We also showed that the sign-tracking behavior in response to rewarding food cues was associated with increased functional connectivity among the fusiform gyrus, occipital lobe, prefrontal cortex, dorsal posterior cingulate cortex, and supramarginal gyrus while participants viewed a cooking video of a sweet dish. This network did not emerge during the viewing of the savory dish during the fMRI scan, suggesting that sign- vs. goal-tracking behaviors and their neural correlates might be specific to characteristics of reward used, in this case, a possible effect of higher sugar content in the sweet video. This specificity between the reward used and the functional network that emerged here might also explain the heterogeneity in results among different studies of Pavlovian conditioning in humans [37,39]. In the observed sign-tracking network, we saw increased functional connectivity in previously established (canonical) networks. Specifically, between the salience and primary visual networks, the sensorimotor and primary visual networks, as well as between the subcortical and medial-frontal canonical networks. Animal studies have shown increased connectivity between the nucleus accumbens, thalamus and amygdala [21], which was not replicated here. However our results agree with other studies in humans that showed the implication of the occipital cortex and supramarginal gyrus in evaluating high-value reward feedback [30]. These regions appear to play a role in salience-modulated attentional bias, food cue reactivity when hungry and activation to cues signaling palatable taste delivery [73–75]. Both prefrontal and posterior cingulate cortex have been linked to increased motivational salience and drug cue reactivity in individuals with substance use disorders [76,77] as well as self-referential processing in healthy individuals [78]. As such, the network identified here suggests that sign-tracking is associated with increased attentional bias and reactivity to food cues as well as increased self-reflection and engagement with the sweet food video during the naturalistic fMRI paradigm.

Understanding the neurobehavioral characteristics of individuals that are more likely to find food cues highly motivating could contribute to designing tailored interventions that could aid in weight management interventions (e.g., increase the incentive salience of cues associated with healthier food options; reduce the presence of food cues in the household environment; shift attentional bias from unhealthy food cues to alternative rewarding cues). Additionally, examining differences on how individuals attribute incentive salience to environmental food cues could aid in treatment approaches that aim to reduce the association between food cues and appetitive behaviors [7]. For instance, during dieting individuals learn to reduce the association between a salient environmental food cue and the appetitive response (e.g., food craving) that leads to overconsumption. However, this extinction process is not always successful and is one of the reasons many people fail to stick to a diet [79], suggesting that differences in Pavlovian learning might play a role the extinction of food cue responsivity [80].

It is important to acknowledge the study limitations. First, we recognize the analytic sample of 47 participants is quite small for the functional connectivity analyses performed. Future studies with larger sample sizes are needed to replicate the present findings. Generally, the machine learning approach implemented here provides a more conservative estimate of the strength of the brain–behavior relationship compared to traditional correlation approaches. However, the ultimate test of the generalizability of our results would be whether the predictive networks identified here would relate to body weight measures in an entirely independent sample. Given that we have not performed this external validation limits the generalizability and replicability of our results. However, it is of note that the functional connectivity network that was positively associated with greater sign-tracking behavior towards a sweet candy reward (M&Ms) emerged only in response to the passive viewing of the “sweet” cooking video and not the “savory”, suggesting that, despite the small sample, there are signs that the brain-behavior associations for sign-tracking might be reward-specific and not generalize to all types of rewards/foods even within the same sample. It is worth noting that although the “sweet” and “savory” cooking videos selected were similar, they were not formally matched or rated for salience and liking, which could potentially confound the results. Second, we found that only WHR, and not BMI, was significantly associated with greater sign-tracking behavior. The BMI association was in the same direction as the WHR but failed to reach statistical significance. This could suggest that BMI on its own might not be an ideal predictor of maladaptive eating behaviors and perhaps other obesity-related indices are better suited to accurately assess the relationship between body composition and maladaptive eating behaviors at earlier stages, even when BMI values are within recommended range [81,82]. In that light, future studies should include more detailed body composition measurements (e.g. BIA, DEXA) and not rely only on BMI and WHR. Third, during the Pavlovian conditioning task, participants were instructed to consume the candy while it was being dispensed. However, some participants were reaching for the candy between trials or were saving multiple candies to eat them all together or stopped eating the candy after they felt full. The amount of candy eaten did not correlate with the ST/GT regression coefficient nor did it differ between the ST and GT groups, but that does eliminate the fact that differences in patterns of eating behavior during the task varied a lot. In this study, we aimed to make the Pavlovian conditioning task more naturalistic and give participants the ability to eat however much and in whatever way they chose to, as they would do in a natural non-laboratory setting. Future studies with smaller samples should standardize that part of the task more to avoid unnecessary variability. Fourth, it is critical to investigate whether the associations between ST vs. GT behaviors and adiposity and functional connectivity observed here are directly related to energy intake and overconsumption. We did not directly address this in our study but future studies should examine relationships with measures of habitual food intake.

In conclusion, this study used a novel food-adapted Pavlovian conditioning task to investigate the neurobehavioral correlates of sign- and goal-tracking in humans. Our findings support that sign-tracking and increased food cue incentive salience are associated with greater adiposity in humans. Additionally, the present study provides new perspectives on the brain functional connectivity that might underlie the sign-tracking phenotype. Increased sign-tracking behavior was associated with higher functional connectivity between brain regions that have been shown to regulate cue responsivity and attentional bias, especially in substance use disorders. These results suggest that differences in Pavlovian conditioning and associative learning could play a role in explaining why some individuals are more susceptible to food cue reactivity and potentially overeating and weight gain. More research is needed to determine whether sign- and goal-tracking behaviors can predict future eating behaviors and weight and whether targeting sign-tracking behaviors in humans can lead to more effective and sustainable weight maintenance.

## Supplementary Material

MMC1

Supplementary material associated with this article can be found, in the online version, at doi:10.1016/j.physbeh.2025.115075.

## Figures and Tables

**Table 1:** Participant Characteristics (*n* = 47).

	Count	Percent

**Sex**		
*Male*	16	34.0
*Female*	31	66.0
**Race/Ethnicity**		
*Asian*	7	14.9
*Black or African American*	2	4.3
*Hispanic or Latino*	1	2.1
*White*	34	72.3
*More than one race*	2	4.3
*Other/Unknown*	1	2.1
**BMI category**		
*Recommended range*	26	55.3
*Overweight*	14	29.8
*Obese*	7	14.9
**Food security status**		
*High or marginal*	38	80.9
*Low or very low*	8	17.0
*Missing*	1	2.1
	Mean (SD)	Min-Max
**Age (years)**	25.3 (5.8)	18–41
**BMI (kg/m^2^)**	25.2 (3.6)	19.1–33.4
**Waist-to-hip ratio**	0.865 (0.046)	0.752–0.986

**Table 2:** Linear regression models testing the relationship between the ST/GT CS value regression coefficient and two adiposity measures (BMI and WHR).

		Outcome: CS value regression coefficient							
	BMI Model					WHR Model			
*Predictors*	*Unstandardized Estimates*	*Standardized Estimates*	*CI*	*p*	*Predictors*	*Unstandardized Estimates*	*Standardized Estimates*	*CI*	*p*
(Intercept)	−0.51	.	−0.98 – −0.05	**0.030**	(Intercept)	−1.64	.	−2.81 – −0.47	**0.007**
BMI	0.01	0.10	−0.01 – 0.02	0.529	WHR	1.48	0.32	0.13 – 2.84	**0.033**
age	0.01	0.32	0.00 – 0.02	**0.039**	age	0.01	0.28	0.00 – 0.02	**0.049**
sex (female)	0.03	0.08	−0.10 – 0.16	0.603	sex (female)	0.07	0.17	−0.06 – 0.20	0.254
pre-task hunger	0.00	0.01	−0.00 – 0.00	0.931	pre-task hunger	−0.00	−0.03	−0.00 – 0.00	0.816
Observations	47				Observations	47			
R^2^	0.141				R^2^	0.222			
R^2^ adjusted	0.059				R^2^ adjusted	0.148			

**Table 3:** High-degree nodes (top 10 %^1^) in the positive ST/GT network for the sweet food show.

Degree	Degree as proportion of network size^2^	Shen 268 atlas node [52]	Region	Canonical Network	MNI Coordinates (x, y, z)
**Positive Network**					
24	0.020	68	R Fusiform Gyrus	VI	25,−45,−12
22	0.018	72	R Occipital Lobe	VAs	21,−64,−9
21	0.017	150	L Prefrontal Cortex	MF	−5,18,46
21	0.017	91	R Dorsal Posterior Cingulate Cortex	SAL	8,−40,48
20	0.017	46	R Supramarginal Gyrus	Mot	58,−29,20
19	0.016	207	L Occipital Lobe	VAs	−26,−63,−12
18	0.015	94	R Hippocampus	SC	36,−15,−18
16	0.013	89	R Dorsal Posterior Cingulate Cortex	Mot	8,−23,45
16	0.013	30	R Premotor Cortex	FP	25,12,49
16	0.013	25	R Supplementary Motor Area	Mot	7,−8,53
15	0.012	161	L Supplementary Motor Area	Mot	−6,−4,48
15	0.012	120	R Cingulate Gyrus	SC	21,−36,23
15	0.012	44	R Precuneus	SAL	7,−57,62
14	0.012	146	L Dorsolateral Prefrontal Cortex	SAL	−27,34,36
14	0.012	28	R Supplementary Motor Area	SAL	6,14,49
13	0.011	73	R Occipital Lobe	VAs	30,−83,21
12	0.010	226	L Dorsal Posterior Cingulate Cortex	SAL	−9,−43,50
12	0.010	214	L Occipital Lobe	VII	−22,−97,−10
12	0.010	213	L Occipital Lobe	VII	−15,−84,−13
12	0.010	149	L Prefrontal Cortex	MF	−39,17,47
12	0.010	136	L Prefrontal Cortex	SAL	−6,18,−22
12	0.010	130	R Brainstem	CBL	10,−19,−31

1 Top 10 % = 22 out of total 220 nodes included in the brain parcellation.

2 Positive network consists of 1210 total edges.

## Data Availability

Data will be made available on request.

## Abbreviations:

AOI: Area of interest
BG: Background
BMI: Body mass index
BOLD: Blood-oxygen level-dependent
CBL: Cerebellar
CPM: Connectome predictive modeling
CS: Conditioned stimulus
DMN: Default mode network
FC: Functional connectivity
FCM: Functional connectivity matrix
fMRI: Functional magnetic resonance imaging
FP: Fronto-parietal
GT: Goal-tracking
MF: Medial-frontal
ms: milliseconds
MNI: Montreal Neurological Institute
Mot: Motor/Somatosensory
PIT: Pavlovian-to-Instrumental transfer
VI: Primary visual
VII: Secondary visual
SAL: Salience
SC: Subcortical
ST: Sign-tracking
UCS: Unconditioned stimulus
VAS: Visual analog scale
VAs: Visual association
WHR: Waist-to-hip ratio
