# Supplementary material for: Sign- vs. goal-tracking is associated with greater adiposity and altered functional connectivity in response to a naturalistic food paradigm
Source: Physiol Behav. Author manuscript; Available in PMC 2025 Oct 3. (PMC12494172; doi:10.1016/j.physbeh.2025.115075)

**Sign- vs. goal-tracking is associated with greater adiposity and altered functional connectivity in response to a naturalistic food paradigm**

**Afroditi Papantoni*, Grace E Shearrer, Lindsey Smith Taillie, Saame Raza Shaikh, Katie A Meyer, Elianna Paninos, Alexxai V Kravitz, Kyle S Burger***

**SUPPLEMENTARY MATERIALS**

*Details for the full Pavlovian-to-Instrumental Transfer (PIT) task*:

The PIT task consists of four parts (**Supplementary Figure 1**):

1. Pavlovian conditioning: We used 4 distinct fractal images (f1, f2, f3, f4) that served as the conditioned stimuli (i.e., ‘fractal CS’), each paired with a different unconditioned stimulus (UCS). Fractal CS 1 (f1) was paired with 2 candies, f2 was paired with 1 candy, f3 was paired with no reward and f4 was paired with no reward + a buzzer sound. F1 and f2 were positive/rewarding CS, f3 and f4 were neutral CS. The CS-UCS pairings were fixed for all participants. At the beginning of each trial, a fixation cross is presented in the middle of the screen for 1.5s. A conditioned stimulus consisting of fractal-like pictures (i.e., ‘fractal CS’) is presented for 3s on the left side of the computer screen, followed by 1.5s two fixation crosses presented on the left and right side of the screen. The unconditioned stimulus (2 candies; 1 candy; no reward; no reward + buzzer sound) is then delivered from the candy dispenser on the right side of the computer screen (opposite of the fractal CS). This is followed by 7.5s of fixation, where the participant consumes the candy and resets their attention and hands.

The first 8 trials presented are a shaping period, where the CS-UCS pairings are presented first in descending order (2 pieces of candy, 1 piece of candy, no reward, no reward + buzzer sound) and then in ascending order (no reward + buzzer sound, no reward, 1 piece of candy, 2 pieces of candy). Following the first 8 shaping trials, all participants complete 64 trials, in which each of the 4 different CS-UCS pairings are presented 16 times in a pseudorandomized sequence. After 32 trials the orientation of the monitor and candy machine are switched, such that the machine delivers candy on the left side of the monitor and the fractal CS appear on the right side of the computer screen. Participants are instructed to observe the fractal CS and the candy delivery and to memorize the pairings. The participants are told to consume that candy by grabbing it with their dominant hand otherwise have their dominant hand placed on the table just below the monitor. They are also told that when the orientation is switched there is no effect on the relationship between the fractal pictures and the candy and that we are simply moving the stimuli around. During the task, eye-tracking data are collected using a pair of Tobii Pro 3 eye tracking glasses (Tobii Pro AB, Stockholm, Sweden). Regular M&M^®^ chocolate candies were used as the rewarding unconditioned stimulus.

1. Instrumental conditioning: At the beginning of each trial a 3s fixation cross is presented in the center of the screen. The goal of the task is to collect rewarding treasure chests and avoid treasure chests that are punishing. To collect a chest, participants have to move an orange dot onto a chest by repeated button presses. Each response moves the dot a fraction of the way towards the chest. 5 button presses (in a 2s response window) are needed to collect the chest. But participants are not informed about the required number of button presses. Participants receive probabilistic feedback. On approach trials, a ‘good’ chest is rewarded with an image of candy in 80% of trials and punished with the candy X’d out in 20% of trials if collected, and vice versa if not collected. On non-approach trials, if a ‘bad’ chest is collected, candy punishment occurs in 80% of trials and is rewarded in 20% of trials, and vice versa if not collected. Participants learn to respond to three ‘good’ (that is, approach) chests and three ‘bad’ (that is, non-approach) chests through trial and error. Participants perform 60–120 instrumental conditioning trials, depending on their performance. To ensure that all of the participants are at comparable performance levels before advancing to the PIT part, a learning criterion is enforced (80% correct choices over 60 trials). Participants are told they will collect the total amount of candy they earn at the end of the session.
2. Pavlovian to instrumental transfer: Participants then perform 72 trials of the instrumental task, as in (b), but with fractal CS (from the Pavlovian task) in the background. No outcomes are presented, but participants are instructed that their choices still count towards the candy at the end of the session. Each of the 6 instrumental chests (3 chests for each of the 2 conditions: instrumental approach/non-approach) is presented with each of the 4 Pavlovian CS a total of 3 times (6 × 4 × 3 = 72 trials), such that instrumental and Pavlovian approach/non-approach are orthogonalized. This is implemented to control for instrumental approach and non-approach tendencies during PIT.
3. Forced choice: In this final part, participants choose one of two sequentially presented fractal CS. Each of the 6 possible fractal CS pairings is presented 4 times in an interleaved, randomized order, yielding a total of 24 trials. Within a trial, CS are presented one at a time for 2s each, one on the left of the monitor (first) followed directly by one on the right (second). This is directly followed by the presentation of the words ‘first image’ or ‘second image’ on the same screen (2s) and the participants are asked to indicate the ‘highest valued image’ as they remember it from part (a) by pressing the left & right arrow buttons. This is followed by a 3s fixation centered in the screen. No feedback is presented. Participants are asked to respond as quickly and accurately as possible once the choice screen appears. Successful learning of the associations in the Pavlovian conditioning task is assessed through the percent of correct choices for each fractal CS pair.

*Quality Control for classifying valid gaze fixations*: The average percent of valid fixations (valid fixations as automatically recorded during data collection by the eye-tracking glasses) was significantly lower in the last 4 trials (13-16) of the task compared to the first 12 (1-12) trials (t = 3.31 , p<0.001) (**Supplementary Figure 2a**). We also performed a repeated measures ANOVA to check the interaction of trial order (1-12 vs. 13-16) and fractal CS type on the percent of valid fixations, but there was no main effect of fractal CS type (p=0.132) or interaction with trial order (p=0.562). Using only the first 12 trials, we further removed bad trials that had a percent valid gaze fixation less than 50% within the last 1.5s of CS presentation. The percent of bad trials removed did not differ significantly by fractal CS type (**Supplementary Figure 2b**). On average, the mean percent of bad trials excluded per CS type across participants was 6.4(11.2)% for fractal CS 1, 7.1(11.5)% for fractal CS 2, 3.1(6.3)% for fractal CS 3, 4.1(7.2)% for fractal CS 4. Percent of gaze fixation on each AOI and subsequent gaze index calculations were performed using only the good remaining trials.

**Supplementary Table 1**. Participant Characteristics by ST/GT group*

|  | **Count (Percent)** | | **p-value** |
| --- | --- | --- | --- |
|  | **Sign-trackers (n=19)** | **Goal-trackers (n=19)** |  |
| **Sex** |  |  |  |
| *Male* | 6 | 7 | 0.732 |
| *Female* | 13 | 12 |  |
| **Race/Ethnicity** |  |  |  |
| *Asian* | 3 | 3 | 0.549 |
| *Black or African American* | 0 | 2 |  |
| *Hispanic or Latino* | 1 | 0 |  |
| *White* | 13 | 13 |  |
| *More than one race* | 1 | 1 |  |
| *Other/Unknown* | 1 | 0 |  |
| **BMI category** |  |  |  |
| *Recommended range* | 8 | 12 | 0.177 |
| *Overweight* | 6 | 6 |  |
| *Obese* | 5 | 1 |  |
| **Food security status** |  |  |  |
| *High or marginal* | 15 | 15 | 1 |
| *Low or very low* | 3 | 4 |  |
| *Missing* | 1 | 0 |  |
|  | **Mean (SD)** | | **p-value** |
|  | **Sign-trackers (n=19)** | **Goal-trackers (n=19)** |  |
| **Age (years)** | 27.5 (5.6) | 22.0 (3.2) | **<0.001** |
| **BMI (kg/m^2^)** | 26.3 (3.7) | 24.6 (3.3) | 0.140 |
| **Waist-to-hip ratio** | 0.881 (0.051) | 0.849 (0.027) | **0.024** |

*9 individuals were classified as intermediates and removed from the post hoc group comparisons


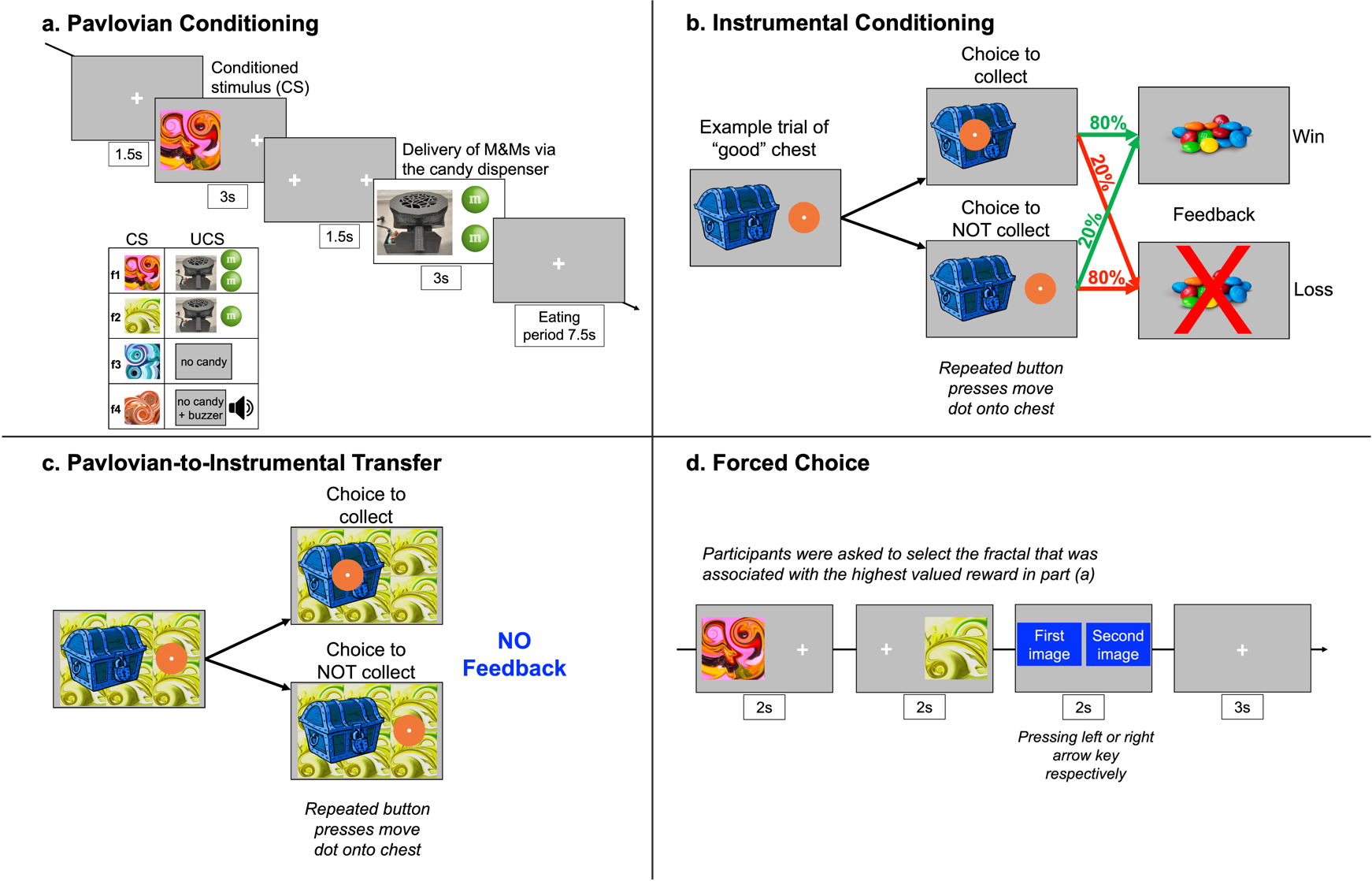
**Supplementary Figure 1**. The PIT Task

The PIT Task [adapted from Schad et al 2020] consisted of four parts: **(a)** Pavlovian Conditioning: Fractal CSs were followed by either food rewards (2 candies, 1 candy) or no rewards (no candy, no candy + buzzer sound). Eye-tracking data were collected during this part. Only data from this part are presented here. **(b)** Instrumental Conditioning: Participants learned to collect three “good” chests and learned to avoid collecting three “bad” chests through trial and error. “Good” chests were collected by 5 button presses and had an 80% chance a win (i.e., earning candy). “Bad” chests had an 80% chance of a loss (i.e., taking candy away) and had to be avoided (i.e., not pressing any buttons). **(c)** Pavlovian-to-Instrumental Transfer: Participants completed the instrumental conditioning task using the same 3 “good” and 3 “bad” chests without any feedback. The background was tiled with the fractal CSs from part (a). **(d)** Forced Choice: Participants were shown multiple pairs of the fractal CSs and were asked to choose the one associated with the highest valued reward.

**Supplementary Figure 2**. Study Visit Timeline

**
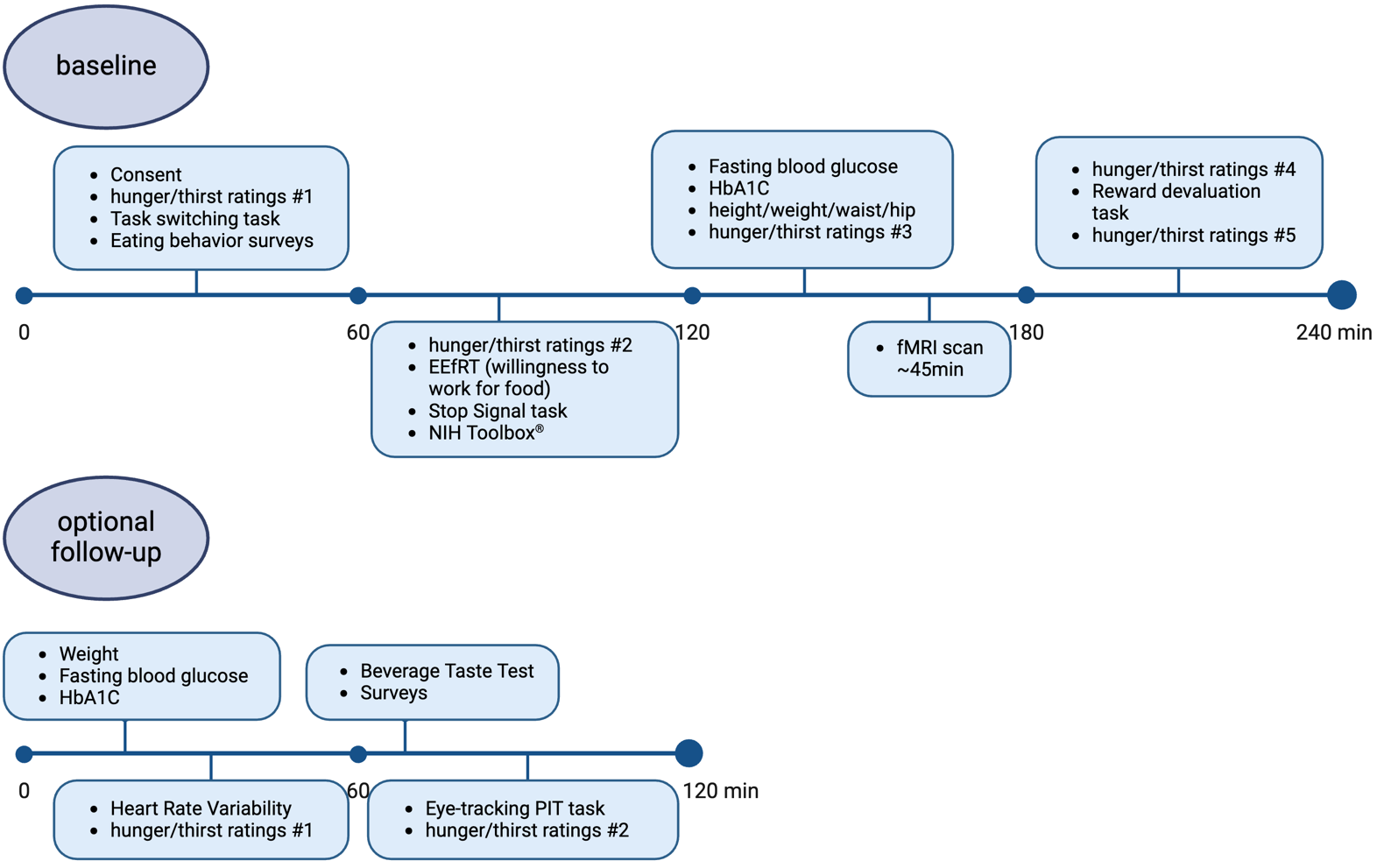
**

**Supplementary Figure 3**. Valid Fixations during eye-tracking in **(a)** early vs. late trials and **(b)** by fractal CS type


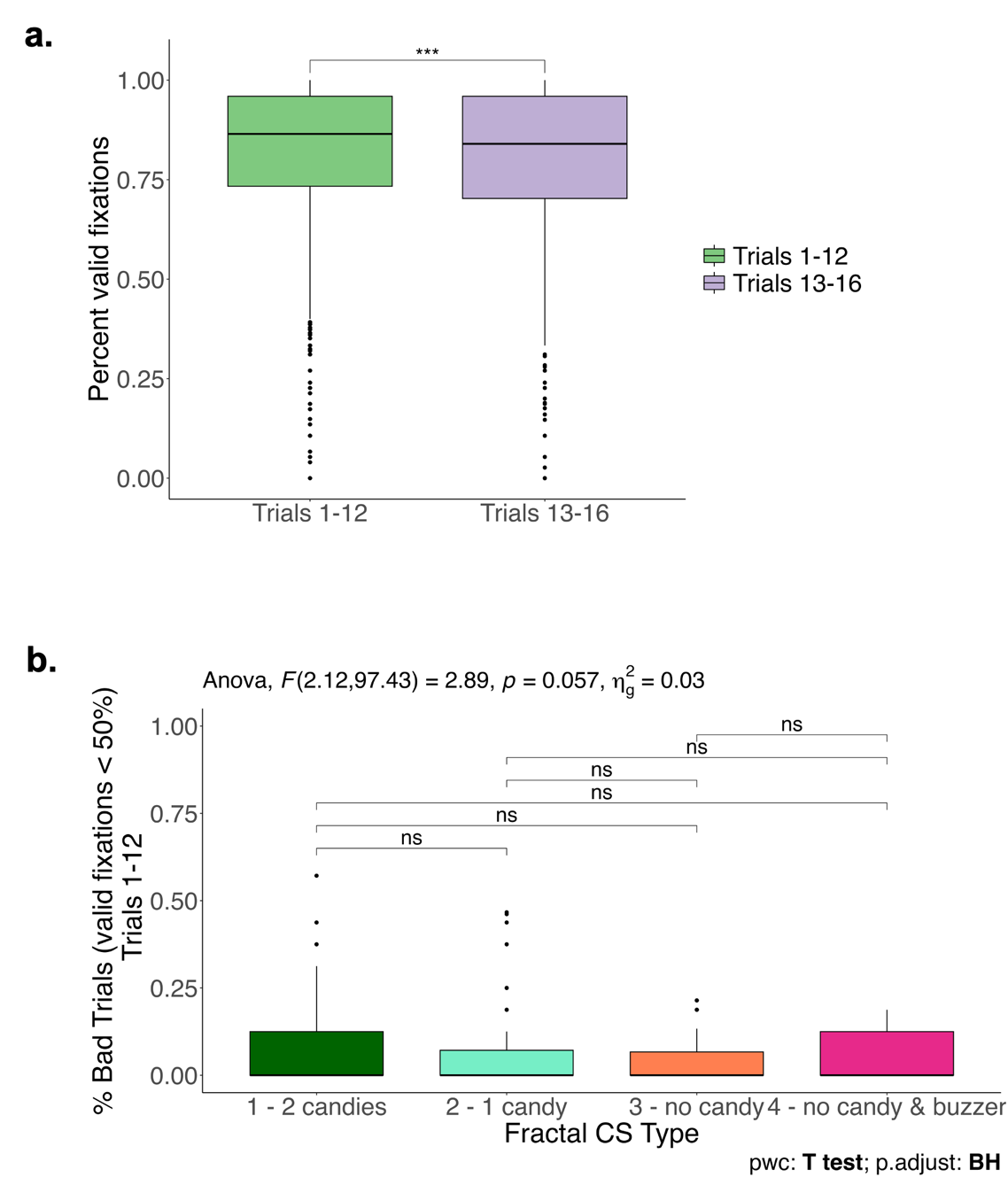


In **(a)** it shows percent valid fixations compared between early (1-12) vs. late (13-16) trials, averaged across all different types of fractal CS. Valid fixations were significantly fewer (t = 3.31, p<0.001) in the late trials suggesting participant fatigue and/or loss of interest in the task. Due to this significant reduction in valid fixations, data from the last 4 trials per fractal CS type were removed from the analysis. There was no significant fractal CS type by trial order (early vs. late) interaction on percent valid fixations (F(3,138)=0.686, p=0.562), further suggesting that reduction of valid fixations was not related to a specific fractal CS but most likely to fatigue. **(b)** Percent of bad trials (calculated as trials with valid fixations < 50%) did not differ by fractal CS type within the first 12 trials of the task (F(2.12, 97.43)=2.89, p=0.057; BH-adjusted post-hoc pairwise p > 0.108).

**Supplementary Figure 4**. Linear relationship between the ST/GT CS value regression coefficient and WHR


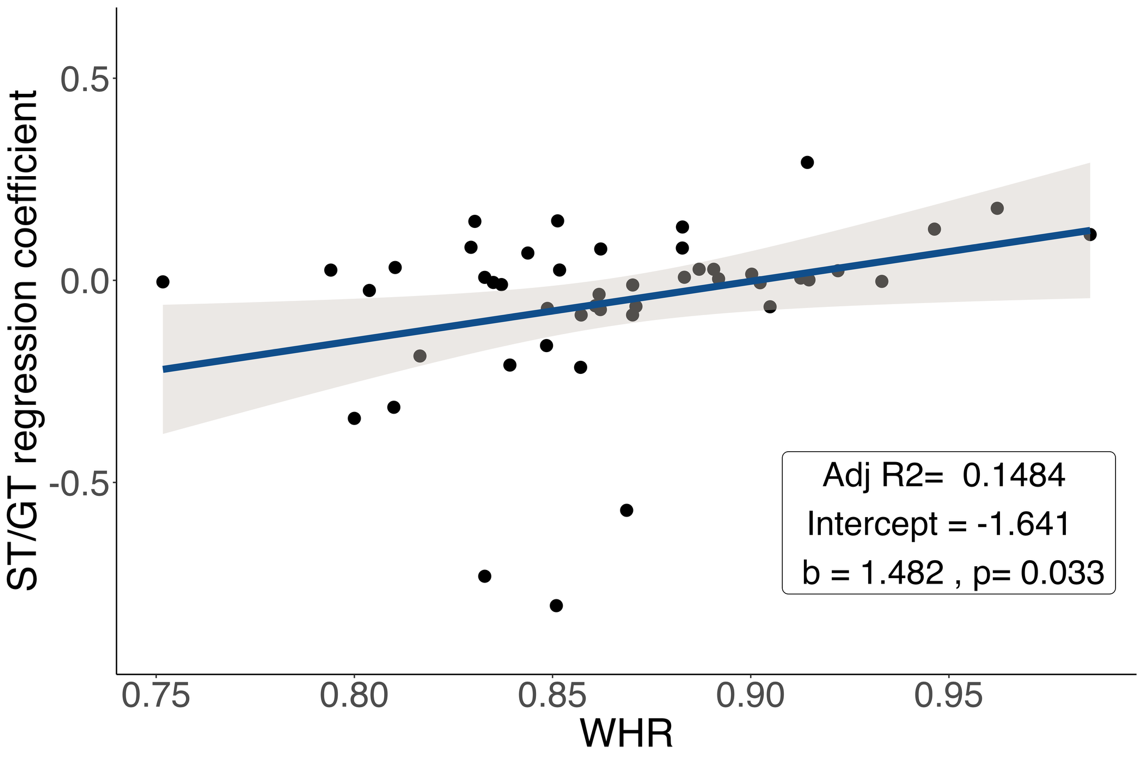


Linear slope showing the positive relationship between WHR and the ST/GT regression coefficient, with higher WHR being linked to more sign-tracking behavior during the Pavlovian conditioning task. Model adjusted for age, sex, and pre-task hunger.

**Supplementary Figure 5**. **(a)** WHR and **(b)** BMI by ST and GT groups


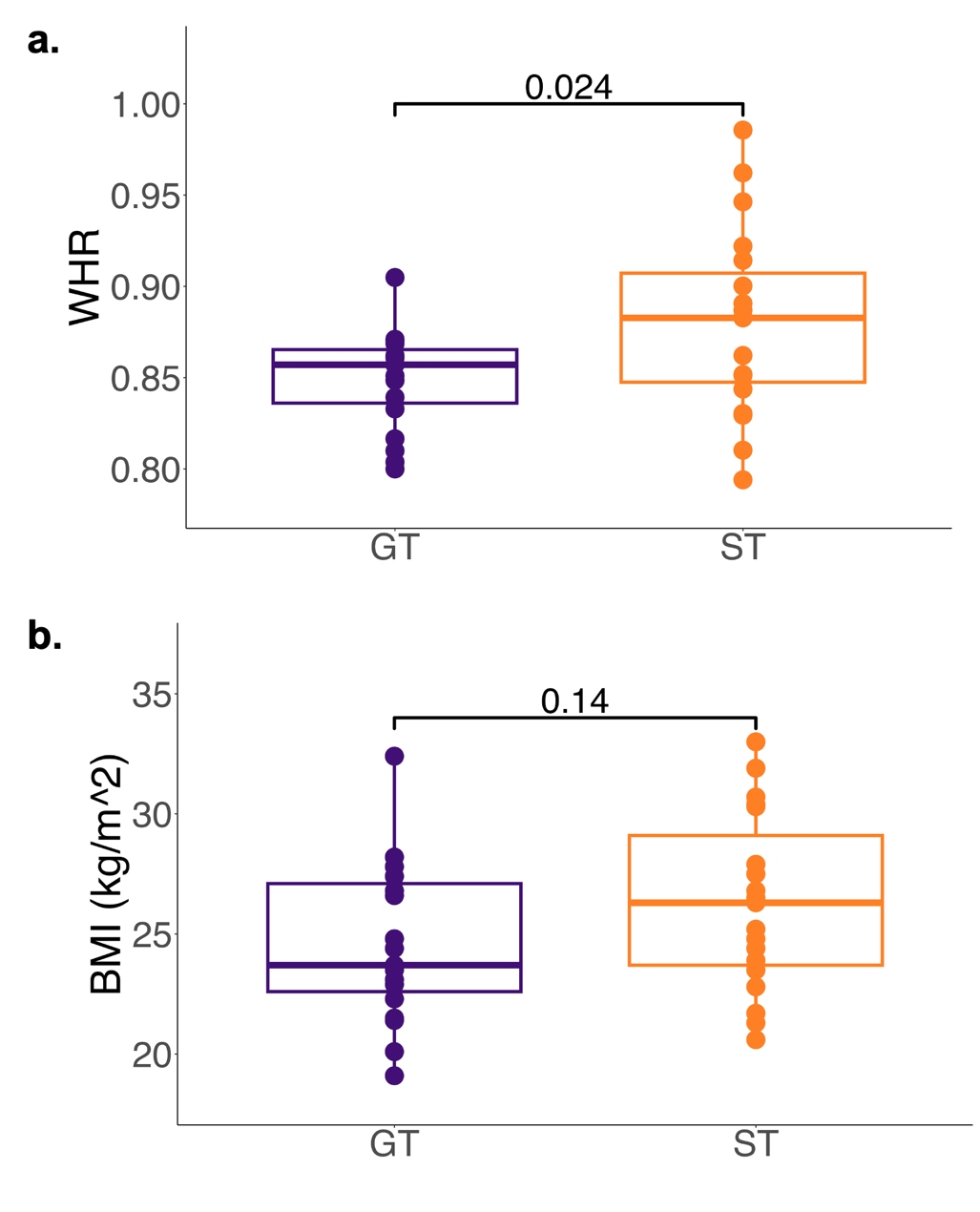

Supplement: MMC1 [file NIHMS2107636-supplement-MMC1.docx]
